# Faunistic Study of the Aquatic Arthropods in a Tourism Area in Northern Iran

**Published:** 2017-05-27

**Authors:** Mansoureh Shaeghi, Hossein Dehghan, Kamran Pakdad, Fatemeh Nikpour, Azad Absavaran, Aioub Sofizadeh, Amir Ahmad Akhavan, Hassan Vatandoost, Abbass Aghai-Afshar

**Affiliations:** 1 Department of Medical Entomology and Vector Control, School of Public Health, Tehran University of Medical Sciences, Tehran, Iran; 2 Depertment of Environmental Chemical Pollutants and Pesticides, Institute for Environmental Research, Tehran University of Medical Sciences, Tehran, Iran

**Keywords:** Aquatic insect, Iran, Mazandaran

## Abstract

**Background:**

Aquatic insects are very abundant and divers groups of insects that are associated with an aquatic or semiaquatic environment in one or more of their life stages. These insects have been, in some cases, well studied because they are vectors of several diseases. This is the first comprehensive faunistic study of aquatic insects from Babol County. The results may provide basic data for further taxonomic and ecological studies of aquatic insects as biological control agent or classification of water quality for the country.

**Methods:**

The specimens were collected using different methods including: D-frame net collector, standard mosquito dipper (350ml), Sweep-Netting and plastic pipette. Sampling carried out in different part of breading places in several times.

**Results:**

During this study a total of 196 aquatic specimens were collected from different habitats and were morphologically identified including 18 families classified in 6 orders: Diptera, Trichoptera, Ephemeroptera, Plecoptera, Hemiptera and Odonata. Babol and Amol district in Mazandaran Province are located in humid climate regions with suitable ecological factors of humidity, moderate temperature and the variety of plant species. There are different species of aquatic insects in different habitats.

**Conclusion:**

The results will provide information for biodeveristy, species richness, their role for biological control as well as calcification of rivers based on abundance of aquatic insects. Therefore the understanding of ecological specifications of aquatic insects could provide a clue for further Arthropod-borne disease control. Additionally aquatic insect could be used for classification of water bodies

## Introduction

Aquatic insects are a very abundant and divers group that inhabits a variety of aquatic environments such as puddles, ponds, lakes, ditches, streams, lakes and other types of breeding places. Despite their importance in aquatic ecosystems, very few insects spend their entire lives submerged in water. Most aquatic insects undergo an aquatic immature stage followed by a terrestrial adult (eg Ephemeroptera, Odonata, Plecoptera, Trichoptera, Megaloptera). Even in cases where both the larvae and adult are aquatic, often the adult can exit the water and/or the pupal stage is terrestrial. Additionally, many species considered are semiaquatic and are only associated with aquatic and semiaquatic vegetation, the water’s surface, or the margins of water habitats (Bouchard 2004).

Although only a small percentage (around 3%) of insects are aquatic, representatives are found in 13 insect orders (Daly 1984, Williams and Feltmate 1992) suggesting that partial aquatic lifestyle might be advantageous to a wide array of insects. Generally, aquatic insects are nymphs/larvae of terrestrial adults (Brown 1987) that spend some time in terrestrial environments during certain stage/s of their life cycle (Jewett 1963). These insects have been, in some cases, well studied because they are vectors of several diseases (Cook 1997), can be environmental quality biosensors (Kashian et al. 2007), and are utilized for understanding aquatic communities and ecosystems (Benke 1979, Waters 1979) and several other areas of ecology such as predator-prey interactions, competition, population dynamics (Resh and Rosenberg 1984). According to the fossil record, aquatic insects appeared in the Triassic (Belayeva et al. 2002), more than 150 MY after the appearance of insects (Gaunt and Miles 2002, Engel and Grimaldi 2004). This fact along with the presence of a tracheal system in nearly all aquatic insects (Chapman 1998), supports the idea that these animals secondarily adapted to living in water (Ross 1967, Resh and Solem 1984, Pritchard et al. 1993).

Some species of aquatic insect are medically important vectors that transmit diseases such as: malaria, dengue, filariasis, yellow fever, anthrax, tularemia, and lyme (Foil 1998). Furthermore few numbers of them have a painful bite that cause dermatological effect on human and animal host (De Villiers 1987). Some of them act as a host of trematods such as: mayfly, dragonfly, damselfly and stonefly (Chae et al. 2000)

Aquatic insects may be used as water indicators of ecosystems as their life span, their comparatively stable mode of life and distinct characters, offers as sorting and identification of these organisms. Many authors have studied the water quality in with aquatic insects are used as indicator of pollution (Krishnamoorthi and Sarkar 1979, Gupta and Paliwal 2007, Agarwal et al. 2008). The various species and population counts of aquatic insect larva present in a stream provide an effective way to monitor the health of the ecosystem. For example, stonefly (Plecoptera) larvae are very sensitive to changes in water quality. On a scale from 0 to 10, with 10 being the most tolerant species, stonefly larva rank between 0 and 2 depending on the specific species (Hilsenhoff 1988).

Some aquatic insects play important roles in mosquito control (James 1966, Ellis and Borden 1970, Pandian et al. 1979). In general, almost all aquatic insect predators prey on mosquito larvae and pupae (Ellis and Borden 1970, Peckarsky 1984), For instance diving beetle *Rhanthus pulverulosus* (Coleoptera, Dytiscidae) and aquatic instars of the order odonata (dragonflies and damselflies) are predators of mosquito larvae and have been observed to ingest mosquito larvae as a part of their natural food assemblages, in particular, may be useful for controlling mosquito larval populations (Sunahara et al. 2002). The backswimmer, *Notonecta undulate* (Hemiptera, Notonectidae) and giant water bug, *Sphaerodema annulatum* and *S. rusticum* (Hemiptera: Belostomatidae), has been shown to efficiently utilize the second instar of mosquito larvae as prey (Ellis and Borden 1970, Murdoch et al. 1984, Blaustein et al. 1995, Blaustein 1998, Pramanic and Raut 2003, Aditya et al. 2004, Aditya et al. 2005). The role of the backswimmer, *Anisops assimilis,* in controlling mosquitoes was recognized as early as 1939, when Graham (Graham 1939) noted some stock troughs with *A. assimilis* were free of mosquitoes, whereas puddles in depressions surrounding the troughs contained “energetic mosquito activity”. However, the predation efficiency of backswimmers on mosquito larvae was found to be container-specific (Lester and Pike 2003). Furthermore, the presence of backswimmers within a water body has been demonstrated to reduce ovi-position rates by adult mosquitoes (Chesson 1984, Blaustein et al. 1995).

Limited numbers of studies have been carried out on the faunistic and ecological aspects of aquatic insects in Iran (Tirgari 1979, Hosseinie 1978, Hosseinie 1992a, Hosseinie 1992b, Hosseinie 1994, Hosseinie 1995a, Hosseinie 1995b, Vafaei et al. 2007, Askari et al. 2009). Babol County is a county of Mazandaran Province in north of Iran (36°33′05″N 52°40′44″E) and located approximately 20 kilometers south of Caspian Sea on the west bank of Babol Rud River and receives abundant annual rainfall. Due to the importance of aquatic insects there is an urgent need for comprehensive studies and publications that are locally available. The present study intends to help fill this gap by providing an overview of the literature published to date on the aquatic insects of Iran especially in Babol County. This is the first comprehensive faunistic study of aquatic insects from Babol County and may provide basic data for further taxonomic and ecological studies of aquatic insects in north of Iran. A further aim of this study was to consider the obtained faunistic results from the ecological aspect.

## Materials and Methods

### Study area

**Fig. 1.**
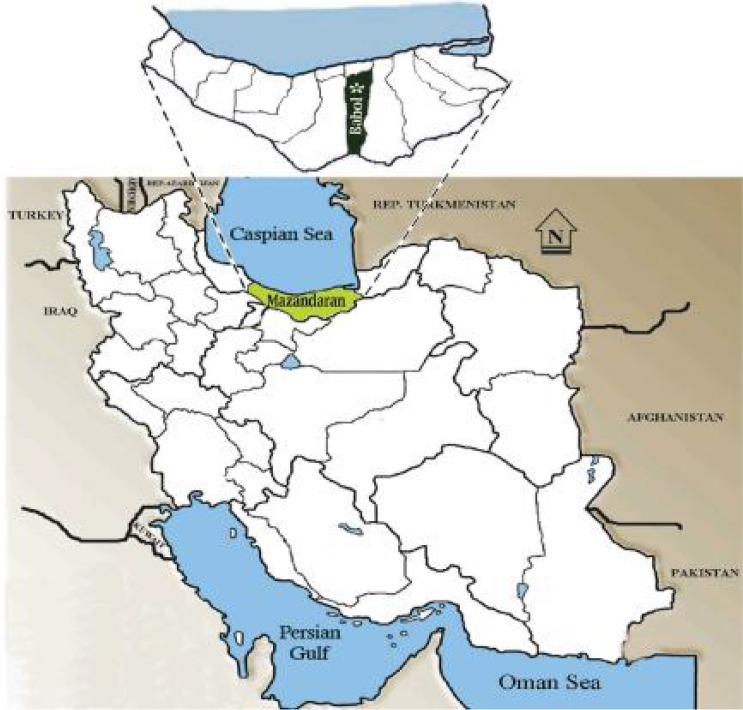
The study areas of sampling and collecting aquatic insects

A descriptive cross sectional study was performed in July 2012 in Babol County, Mazandaran Province. This province, located in the north of Iran and southern shores of the Caspian Sea (36° 33′ 56.16″ N, 53° 3′ 31.68″ E) (Fig. 1). Climatic characteristics of the Caspian Sea Coasts are almost Mediterranean. Generally its climate is temperate and humid and approximately is equilibrium with hot and cold throughout the year (heat and cold, 5 °C and 25 °C). Considering various factors which influence the climate of the province are in the area, there are two types of weather: temperate plains and mountains (temperate and cold).

In this study, rivers, rice fields, and the stagnant water in different parts of province were selected as sites for sampling and collecting of aquatic insects.

### Sampling methods

The specimens were collected using different methods including: D frame net-collector, standard mosquito dipper (350ml), Sweep-Netting and plastic pipette. Sampling carried out in different part of breading places in several occasions. The samples were collected, transferred to individual jars containing some water obtained from their habitat. Subsequently they were put in glass vials contained 70% ethylic alcohol.

The date and location of sampling were written on the label and stick on the vials. All samples were sent to School of Public Health, Tehran University of Medical Sciences, where the author identified the specimens using the keys of aquatic insects based on Guide to Aquatic Invertebrate Families of Mongolia 2012 and other relevant systematic keys. In this study we used stereo-typed microscope and microscope to identification of samples. The results were recorded on a data sheet based on the order and family and number of its. All of the photos are original.

## Results

During this study 196 aquatic insects (mature and immature forms) were collected from different habitats (slow moving and turbulent rivers, shallow streams, temporary pond, andrice field that its information is shown in Table 1.

**Table 1.** Collected insect orders from different habitats, Babol and Amol, Mazandaran Province, 2012

**Habitat**	**Orders**
**Rice-field**	Ephemeroptera Diptera _____ _____
**Slow moving river**	Ephemeroptera Diptera Hemiptera Trichoptera
**Temporary pond**	Ephemeroptera Diptera Hemiptera _____
**Shallow stream**	Ephemeroptera _____ Hemiptera Trichoptera Odonata
**Turbulent river**	_____ Diptera Hemiptera Trichoptera Plecoptera

**Table 2.** Biodiversity of families in 6 orders of aquatic insects collected in Babol and Amol, Mazandaran Province, 2012

**Order**	**No. of Families**
**Diptera**	5
**Odonata**	4
**Hemiptera**	4
**Ephemeroptera**	3
**Plecoptera**	1
**Trichoptera**	1

Collected aquatic samples were identified by morphologic traits with using valid entomological keys (William Bouchard 2012, Subramanian and Sivaramankrishnan 2007).

**Fig. 2.**
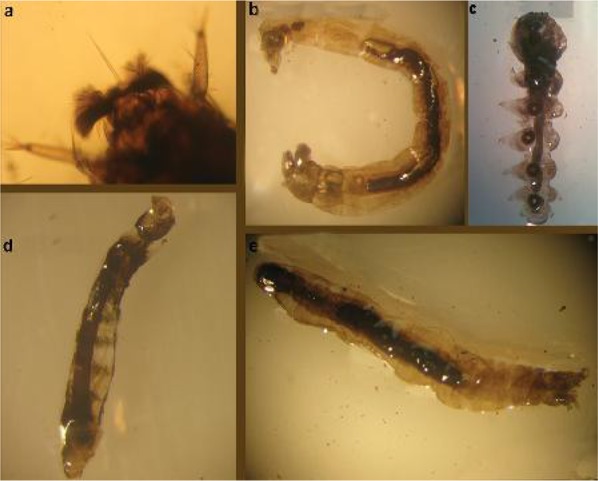
Order Diptera: a. Culicidae, b.Chironomidae, c.Blephariceridae, d.Simulidae, e.Tipulidae

**Fig. 3.**
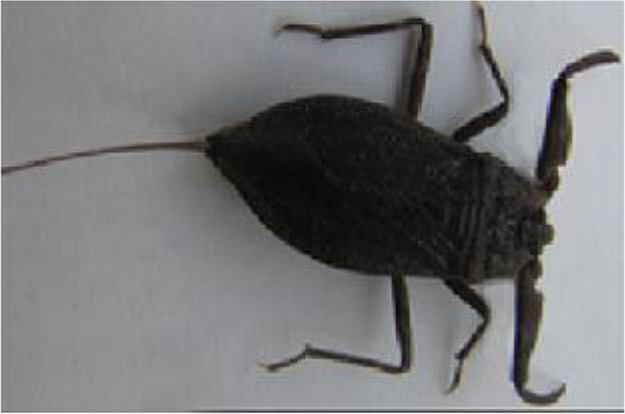
Order Hemiptera: Nepidae

**Fig. 4.**
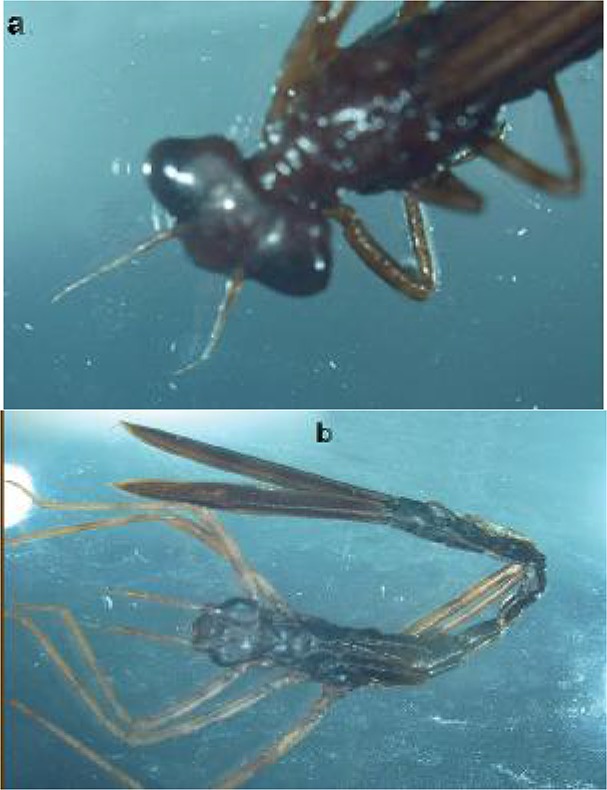
Order Odonata: a.Coenagrionidae, b.Caloepterygidae

**Fig. 5.**
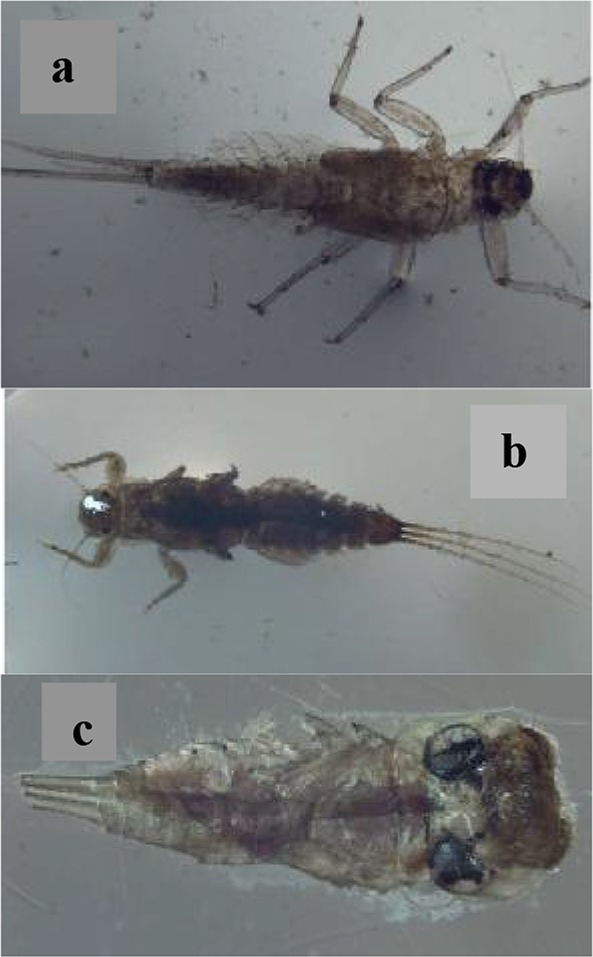
Order Ephemeroptera: a.Baetidae, b.Caenidae, c.Heptagenidae

**Fig. 6.**
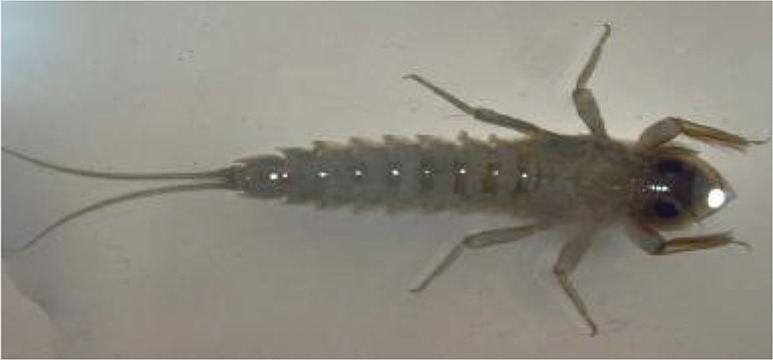
Order Plecoptera: Taeniotorygidae

**Fig. 7.**
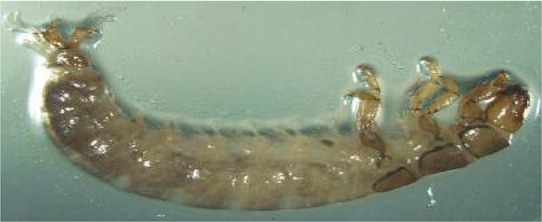
Order Trichoptera: Helicopsychidae

These aquatic insects after identifying represented 18 families belonged to 6 orders: Diptera, Trichoptera, Ephemeroptera, Plecoptera, Hemiptera and Odonata (both sub orders Zygoptera and Anisoptera). Diptera with 5 families: Culicidae, Tipulidae, Chironomidae, Simulidae and Blephariceridae (Fig. 2A–E), Hemiptera with four families: Nepidae, Corixidae, Notonectidae and Gerridae (Fig. 3), Odonata with four families: Calepterygidae, Coenagrionidae, Lestidae and Aeshnidae (Fig. 4A, B), Ephemeroptera with three families: Baetidae, Heptagenidae and Caenidae (Fig. 5A–C), Plecoptera and Trichoptera with one family Taeniopterygidae and Helicopsychidae respectively (Fig. 6 and Fig. 7). In this study Diptera had the most and Trichoptera associated with Plecoptera had the least families.

**Fig. 8.**
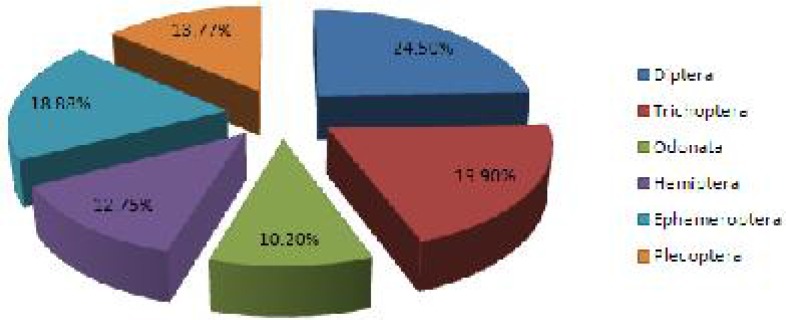
Percentage of aquatic insects orders, Babol and Amol, Mazandaran Province, 2012

From 196 identified aquatic insects 48 (24.5%) belonged to five families of Diptera, 39(19.9%) from Tricoptera, 37(18.88%) from 3 families of Ephemeroptera, 27 (13.77%) from Plecoptera, 25(12.75%) from 4 families of Hemiptera and 20(10.2%) from 4 families of Odonata (Fig. 8).

Helicopsychidae family and (Blephariceridae, Caenidae, Lestidae and Aeshnidae) allocated the most (19.9%) and the least (0.51) frequency of families in our study respectively (Table 3).

**Table 3.** Aquatic insects families frequency collected from Babol And Amol, Mazandaran Province, 2012

**Order**	**Sub.Order**	**Family**	**No. (%)**	**Total (%)**
**Diptera**		Culicidae	26 (13.27)	48 (24.5)
		Simulidae	10 (5.1)	
		Chironomidae	7 (3.58)	
		Tipulidae	4 (2.04)	
		Blephariceridae	1 (0.51)	
**Trichoptera**		Helicopsychidae	39 (19.9)	39 (19.9)
**Ephemeroptera**		Baetidae	34 (17.35)	37 (18.88)
		Heptagenidae	2 (1.02)	
		Caenidae	1 (0.51)	
**Plecoptera**		Taeniopterygidae	27 (13.77)	27 (13.77)
**Hemiptera**		Corixidae	12 (6.12)	25 (12.75)
		Nepidae	6 (3.06)	
		Gerridae	4 (2.04)	
		Notonectidae	3 (1.53)	
**Odonata**	Zygoptera	Caloepterygidae	15 (7.65)	20 (10.2)
		Coenagrionidae	3 (1.53)	
		Lestidae	1 (0.51)	
	Anisoptera	Aeshnidae	1 (0.51)	
**Total**				196 (100)

### Order

#### Diptera

We find five families in Diptera order which described in bellow: (Bouchard 2012)

#### Culicidae

Common Name: Mosquitoes

Habitat: Mosquito larvae occur in standing or still water of lakes, ponds, marshes, temporary pools, and streams. The larvae are planktonic.

Size: Small to medium (4–18mm)

Characteristics: Head sclerotized, rounded, and clearly separate from the thorax, labrum with brushes of setae; mandibles moving against each other on a horizontal plane, thoracic segments fused and swollen, wider than abdomen, Prolegs absent, eighth segment usually bearing a respiratory siphon.

#### Chironomidae

Common Name: Non-Biting Midges

Habitat: Chironomids are found in every aquatic habitat from small seeps to large rivers and from temporary pools to deep lakes. They occur in soft sediment, on rocks, in and around vegetation, in snags, and just about any other habitat.

Size: Small to large (2–30mm)

Characteristics: Head sclerotized, rounded, and clearly separate from the thorax, body elongate and worm-like, mandibles moving against each other on a horizontal plane, two pairs of ventral prologs (one on prothorax and one at the terminal end), prologs terminate in a series of hooks.

#### Tipalidae

Common Name: Crane Flies

Habitat: Tipulid larvae can be found in a variety of habitats such as streams, ponds, and marshes. They can be found under rocks, in sand, snags, leaf packs, and algal mats.

Size: Small to large (3–60mm)

Characteristics: Much of rounded head capsule present or reduced to only a few rods, head capsule completely or partially retracted into thorax, mandibles moving against each other on a horizontal plane, usually with ventral welts, terminal segment usually with two spiracles, spiracular disc usually surrounded by lobes or projections of varying numbers or shapes.

#### Blepharicerida

Common Name: Net-Winged Midges

Habitat: Blepharicerid larvae are restricted to cool, fast-flowing streams and waterfalls. They are found attached to rocks in areas of fast flow.

Size: Small to medium (5–12mm)

Characteristics: Head fused with thorax and first abdominal segment, mandibles moving against each other on a horizontal plane, 6 abdominal segments with deep constrictions between segments, ventral suckers on first 6 segments, gill tufts present ventrally.

#### Simuliidae

Common Name: Black Flies, Buffalo Gnats

Habitat: Black fly larvae occur in streams and rivers in areas of moderate to fast current. They are found attached to rocks, logs, vegetation, or any other solid substrate in the current.

Size: Small to medium (3–15mm)

Characteristics: Head sclerotized, rounded, and clearly separate from thorax, pair of labral fans (“mouthbrushes”) usually present, mandibles moving against each other on a horizontal plane, proleg present ventrally on prothorax, posterior 1/3 of abdomen swollen, abdomen terminates in a ring of hooks.

### Order

#### Hemiptera

Aquatic hemipterans are unlike most aquatic taxa in that the adults and larvae occupy the same habitat. Aquatic and semiaquatic Hemiptera can be separated into two groups based on their antennal morphology and the habitat in which they are generally found. Some Hemiptera are primarily aquatic and can be recognized by the possession of antennae that are shorter than the head and concealed below the eye. One exception is the Gelastocoridae, which are riparian and possess short antennae. The truly aquatic species are usually found under water, but many possess wings, which allow movement between water bodies. In contrast, most semiaquatic species of Hemiptera have antennae longer than their heads and can be found on the water’s surface or at the water’s margin.

Although some taxa are primarily aquatic, most Hemiptera do not rely heavily on dissolved oxygen in the water, but instead obtain oxygen from the atmosphere.

We find four families in Hemiptera order which described in bellow: (Bouchard 2012)

#### Corixidae

Common Name: Water Boatmen

Habitat: Corixids are found in areas of standing or slow flowing water in ponds, lakes, marshes, streams, and rivers.

Size: Small (3–11mm)

Characteristics: Antennae shorter than head, concealed below eye, beak broad and triangular without distinct segments; fore tarsus scooplike and edged with setae.

#### Nepidae

Common Name: Water Scorpions

Habitat: Nepids can be collected in ponds, marshes, and streams in areas of calm water. They are usually found at the vegetated margins of these water bodies.

Size: Medium to large (15–45mm) –not including respiratory tube.

Characteristics: Body cylindrical or oval and flattened (not shown but when body is oval it is superficially similar to Belostomatidae), antennae shorter than head, concealed below eye, beak cylindrical, fore legs raptorial, mid and hind legs long and slender, abdomen terminating in an elongate breathing appendage.

#### Gerridae

Common Name: Water Striders

Habitat: Gerrids are generally found on the surface of the water in ponds, lakes, marshes, streams, and rivers.

Size: Small to medium (3–18mm)

Characteristics: Body shape variable, antennae longer than head, beak cylindrical, claws of protarsus inserted before apex, metafemur extends well beyond tip of abdomen.

#### Notonoctidae

Common Name: Backswimmers

Habitat: Notonectids most commonly occur along vegetated margins of lakes and ponds and in marshes. They are also sometimes found in the pools and backwaters in streams and rivers. Notonectids can also be found in temporary pools and ditches.

Size: Small to medium (5–15mm)

Characteristics: Body cylindrical, antennae shorter than head, concealed below eye, beak cylindrical, hind legs oar-like, hind tarsal claws inconspicuous

### Order

#### Odonata

We find four families in Odonata order which described in bellow: (Bouchard 2012)

#### Calopterygidae

Common Name: Broad-Winged Damselflies

Habitat: These damselflies are most commonly found at the edges of streams with slow flowing water where they cling to root masses, overhanging grasses, and twigs.

Size: Large (30–40mm)

Characteristics: Antennal segment 1 longer than remaining antennal segments put together, prementum with diamond shaped medial cleft, prementum and palpal setae absent, middle gill shorter than lateral gills, gills triangular in cross section, no veins visible in gills.

#### Coenagrionidae

Common Name: Narrow-Winged Damselflies

Habitat: Narrow-winged damselflies are found in a wide range of habitats including ponds and flowing waters. These damselflies are most common in vegetation at the margins of lakes and in wetlands. Some species are found in streams clinging to rocks and vegetation.

Size: Medium to large (15–32mm)

Characteristics: Most are slender like other damselfly larvae (although some are short and stocky), all antennal segments are approximately the same length, prementum triangular and stout without medial notch, usually 3–5 premental setae on each side of midline, 1–7 raptorial setae on each palpal lobe, palpal lobes terminating in 1–2 hooks, all gills the same length, veins in gills radiate diagonally from medial line.

#### lestidae

Common Name: Spread-Winged Damselflies

Habitat: These damselflies are most common in small ponds, bogs, wetlands, and sometimes in slow weedy streams.

Size: Large (22–38mm)

Characteristics: These are long slender damselflies, all antennal segments similar, prementum with small triangular notch, prementum stalked and spoon shaped, 4–8 premental setae present, palpal lobes with 3–5 raptorial setae and trifid, all gills of similar length, veins visible in gills and perpendicular to medial line.

#### Aeshnidae

Common Name: Darner Dragonflies

Habitat: Darner dragonflies are most commonly collected in vegetation along the edges of lakes and ponds. They can also be found in some streams under logs and stones or in snags.

Size: Large (30–62mm)

Characteristics: Prementum and palpal lobes are flattened, 6–7 antennal segments present with all segments of similar size and shape.

### Order

#### Ephemeroptera

We find three families in Ephemeroptera order which described in bellow: (Bouchard 2012)

#### Baetidae

Common Name: Primitive Minnow Mayflies

Habitat: Primitive minnow mayflies can be found in vegetation along large rivers, in the riffles of small streams, in seeps, in swamps, and in ponds.

Size: Small to large (6–20mm)

Characteristics: Antennae less than 2x the width of head, maxillae without pectinate spines, gills usually present on abdominal segments 1–7, gills usually oval, long setae present on caudal filaments (present on both sides of terminal filament and only on the inner side of the cerci).

#### Heptageniidae

Common Name: Flathead Mayflies

Habitat: Flathead mayflies are most common in slow to fast flowing streams where they occur on the surface of rocks, logs, vegetation, and leaves. HEMEROPTERA

Size: Small to large (5–20mm)

Characteristics: Body, head, and legs (femora) flattened, mouthparts not visible from dorsal view, gills present on abdominal segments 1–7, only short setae present on caudal filaments.

#### Caenidae

Common Name: Small Square-Gill Mayflies

Habitat: Caenid may fly larvae occur in streams in areas of slow current, at the edges of lakes, and in wetlands.

Size: Small (2–8mm)

Characteristics: Gills on abdominal segment 1 vestigial (small and finger-like), gills on abdominal segment 2 square operculate (plate-like) and covering succeeding gills, operculate gills touch or overlap at midline, fringed gills present on abdominal segments 3–6, setae on caudal filaments restricted to apex of each annulation.

### Order

#### Plecoptera

Of this order we collected only one family: Taeniopterygidae which described in bellow: (Bouchard 2012)

#### Taeniopterygidae

Common Name: Giant Stoneflies

Habitat: These large stoneflies are most commonly found in small swiftly flowing streams. They are found in areas of swift current in leaf packs and snags.

Size: Large (15–70mm)

Characteristics: Larvae are large and dark brown, finely branched gills are present on all 3 thoracicsegments and abdominal segments 1–2.

### Order

#### Tricoptera

in this study we collected only one family in Tricoptera order: Helicopsychidae which described in bellow: (Bouchard 2012).

#### Helicopsychidae

Common Name: Snail Case-Maker Caddisflies

Habitat: These caddisflies are most commonly found in streams with sand deposits. They are also found on wave-swept shores of lakes. Snail casemaker caddisflies are usually attached to rocks and logs.

Size: Small (8mm) –the case is usually about the size of a pea.

Characteristics: Body curled, all three thoracic segments with sclerotized dorsal plates, stout setae present on anterior edge of pronotum, prosternal horn absent, branched gills present on anterior abdominal segments, anal claw comb-shaped (with many small teeth), case shaped like a snail shell.

## Discussion

Given that Babol and Amol district in Mazandaran Province are in humid climate regions with suitable ecological factors humid, moderate temperature and the variety of plant species where there are different species of aquatic insects in different habitats (slow moving and turbulent rivers, shallow streams, temporary pond, and rice fields, these areas are the appropriate places for the growth of aquatic insects and sample collection. In the current study, the most prevalent collected sample was Diptera with five families (24.5%) and Trichoptera, Ephimeroptera, Plecoptera, Hemiptera and Odonata seted in latter rank respectively.

In a study conducted in Yamuna in summer, as well as the results of our study the most abundant order were Diptera and Hemiptera, Coleoptera and Odonata seted in latter rank respectively (Gapta and Paliwal 2010).

In a study conducted in USA Ephimeroptera, Odonata, Plecoptera, Hemiptera, Megaloptera, Coleoptera, Trichoptera and Diptera presented in eight major order of aquatic insects (Reese Voshell 2009). We collected five order of this eight order in clouded: Diptera, Trichoptera, Ephimeroptera, Plecoptera, Hemiptera and Odonata.

In a study conducted in Nigeria abundance of order of aquatic insecs were as follows:

Ephimeroptera >Odonata >Coleoptera > Diptera >Pelicoptera >Tricoptera (Ohiokhioya 2009).

### Ephemeroptera (Mayflies)

Mayfly larvae are found in a variety of locations including lakes, wetlands, streams, and rivers, but they are most common and diverse in lotic habitats. They are common and abundant in stream riffles and pools, at lake margins and in some cases lake bottoms. Most of them eat plant material, either by scraping algae or collecting small pieces of detritus from the bottom. They have incomplete metamorphosis. All mayfly larvae are aquatic and breathe dissolved oxygen by means of gills on the abdomen with terrestrial adults. In most mayfly species the adult only lives for 1–2 days. Consequently, the majority of a mayfly’s life is spent in the water as a larva. The adult lifespan is so short there is no need for the insect to feed and therefore the adult does not possess functional mouthparts so never eat. Mayflies are often an indicator of good water quality because most mayflies are relatively intolerant of pollution. Mayflies are also an important food source for fish (Reese Voshell 2009, Bouchard 2012).

In this study, we were founded may flies in rice-field, slow moving river, temporary pond and shallow stream, but don’t fonded of turbulent river. Of this order we collected 3 families: Baetidae (91/5%), Heptagenidae (5/5%) and Caenidae (3%).

### Caddisflies (Tricoptera)

Trichoptera is the largest order of insects in which most members are truly aquatic. Trichoptera are close relatives of butterflies and moths (Lepidoptera) and like Lepidoptera, Larvae of different caddisflies live in a wide variety of flowing and standing waters. They also have a wide range of feeding habits, including scraping algae, collecting fine particles of detritus from the bottom or from the water, shredding dead leaves, and preying on other invertebrates. They breathe dissolved oxygen by means of gills and their overall body surfaces have complete metamorphosis and remain in the water for the pupa stage. Caddisflies have the ability to spin silk. This adaptation may be largely responsible for the success of this group. Silk is used to build retreats, to build nets for collecting food, for construction of cases, for anchoring to the substrate, and to spin a cocoon for the pupa. Almost all caddisflies live in a case or retreat with the exception of Rhyacophilidae. Caddisflies are important in aquatic ecosystems because they process organic material and are an important food source for fish. This group displays a variety of feeding habits such as filter/collectors, collector/gatherers, scrapers, shredders, piercer/herbivores, and predators. Caddisflies are most abundant in running (lotic) waters. Like Ephemeroptera and Plecoptera, many Trichoptera species are sensitive to pollution but a few kinds are somewhat tolerant of moderate levels of pollution. (Reese Voshell 2009, Bouchard 2012).

In this study, we wre founded Trichoptera in slow moving river, shallow stream and Turbulent river.

### Plecoptera (Stoneflies)

Plecoptera have incomplete metamorphosis. They larvae are almost exclusively found in running waters and they reach their greatest diversity in small cold streams. They are generally associated with coarse substrates such as cobble, leaf packs, and large woody debris. Plecoptera are the most sensitive order of aquatic insects and many species are restricted to habitats with high levels of dissolved oxygen. Some have gills on their thorax, but others just obtain dissolved oxygen all over their body. Some feed on plant material, either by shredding dead leaves and other large pieces of detritus, while others are predators.When a stonefly larva is ready to emerge as an adult, it crawls out of the water and sheds the larval skin or exoskeleton. Compared to the length of the immature stage (6 months to 3 years), the adult life span is short and usually lasts only 1–4 weeks.

Many adult stoneflies exhibit an interesting behavior of drumming to locate mates. A male will usually initiate drumming by tapping its abdomen on the substrate. A female that perceives the vibrations will then drum a response. By moving toward each other while periodically stopping to drum, males and females are able to locate each other. In order to insure that males and females of the same species find each other, each species has a unique drumming pattern. The most unusual feature of this group is that some kinds are programmed to emerge only during the coldest months, hence, they are called the winter stoneflies. Like Ephemeroptera and Tricoptera, Almost all of the stoneflies are sensitive to pollution.

In this study, we found Plecoptera in turbulent river but didn’t find in rice-field, slow moving river, temporary pond and shallow stream.

### Hemiptera (True Bugs)

Most of the true bugs live on land, but the aquatic kinds are most common in the shallow areas around the edge of standing waters. Both the adults and the larvae of the aquatic kinds live in the water. Both stages are usually found on submerged aquatic plants. Almost all of them are predators. They breathe oxygen from the air, either by taking a bubble underwater or by sticking a breathing tube up into the air. They have incomplete metamorphosis.

Most kinds are tolerant of pollution. True bugs have a sharp beak that they stick into the body of their prey, and then they pump in poison to kill their prey, after which they suck out the body fluids. Some of the larger kinds feed on small fish and tadpoles (Reese Voshell 2009).

Due to their ability to utilize atmospheric oxygen, Hemiptera are often able to exist in water bodies with low levels of dissolved oxygen (Bouchard 2012).

In this study, we found Hemiptera in turbulent river, slow moving river, temporary pond and shallow stream, but don’t fonded in rice-field. Of this order we collected 4 families: Corixidae (48%), Neppidae (24%), Geridae (16%) and Notonectidae (12%).

### Odonata (Dragonflies and Damselflies)

Most people are familiar with large dragonflies observed flying over ponds, marshes, and fields. Less well known are the aquatic larvae of these insects. They have incomplete metamorphosis. Odonates are most abundant and diverse in lentic (standing) waters, but many species also occur in lotic (flowing) waters. Larvae breathe dissolved oxygen with gills, which are located either inside the rear portion of the abdomen (dragonflies) or on the end of the abdomen (damselflies). All adult and larval odonates are predatory. Most Odonata larvae are sit-and-wait predators, which means they remain motionless until an insect or small fish approaches the larva. When a prey item comes close, the larva rapidly extends its labium (lower lip) and grasps the prey with its jaw-like palps. The most unusual feature of this group is the way the larvae catch their food with an elbowed lower lip, which they can shoot out in front of the head. The adults feed on arthropods such as other insects (including other dragonflies and damselflies) and spiders. Adult odonates, especially dragonflies, are strong fliers and catch much of their prey on the wing. Many kinds are fairly tolerant of pollution, but some kinds only live in unique habitats, such as bogs high in the mountains.

In this study, we founde odonata only in shallow stream. We found Zygoptera and Anisoptera suborder. From suborder Zygoptera we found 3 Families: Caleopterygidae (79%), Coenagrionidae (15/7%) and Lestidae (5/2%), ad in Anisoptera suborder we founded only one sample of Aeshnidae.

#### Diptera (True Flies)

Diptera considered aquatic have aquatic larvae and pupae with terrestrial adults. Many other aquatic insects are also commonly referred to as “flies” (eg mayflies, dragonflies, stoneflies, caddisflies, alderflies, fishflies), but these taxa are not true flies as they do not belong to the order Diptera. When referring to true flies or Diptera with their common names, the word “fly” is separate (eg crane fly, black fly, moth fly dance fly, flower fly). In contrast, common names for non-dipteran taxa are one word. The true flies are extremely important in aquatic food webs and often are the most diverse and abundant macroinvertebrate taxon collected in many freshwater habitats. They have a wide range of feeding habits, including scraping algae, collecting fine particles of detritus from the bottom or from the water, shredding dead leaves, and preying on other invertebrates. Diptera inhabit a wide range of habitats and some taxa are extremely tolerant and occur in heavily polluted water bodies. They breathe dissolved oxygen by means of gills and their verall body surface. True flies have complete metamorphosis and remain in the water for the pupa stage. The most distinctive feature of this group is their ecological diversity. Some kinds live in the cleanest habitats (eg swift, cool, mountain streams), while others live in some of the harshest natural habitats on the earth (e.g., arctic tundra ponds, geothermal springs, alkaline lakes, mucky swamps). They have equally diverse responses to pollution, with some kinds being exceptionally sensitive, while other kinds endure the worst imaginable water quality (eg raw sewage or acid mine drainage). Some true flies can be a nuisance due to their blood feeding behaviors (Bouchard 2012).

In this study, we found Diptera in rice-field, slow moving river, temporary pond and turbulent river, but didn’t find in shallow stream. From this order we collected 5 families: Culicidae (54/1%), Simulidae (20/8) Chironomidae (14/5%), Tipulidae (8/3%) and Blepharieridaec (2%).

## Conclusion

Aquatic insects play an important role for biological control of larvae and adults of mosquitoes. On the other hand some of them are vectors of human and animal diseases, for example in Iran several species belong to Anopheline including *Anopheles culicifacies s.l.*, *An. stephensi*, *An. dthali*, *An. fluviatilis s.l.*, *An. superpictus*, are known to be the malaria vectors (Naddaf et al. 2003, Doosti et al. 2003, Oshaghi et al. 2003, Oshaghi et al. 2006, Vatandoost et al. 2006, Davari et al. 2007, Hanafi-Bojd et al. 2011, Mehravaran et al. 2011, Oshaghi et al. 2011, Vatandoost et al. 2011, Hanafi-Bojd et al. 2012a, b, c, Vatandoost and Abai 2012, Soleimani-Ahmadi et al. 2012a,b, Vatandoost and Hanafi-Bojd 2012). Therefore the understanding of ecological specifications of aquatic insects could provide a clue for further Arthropod-borne disease control. Additionally aquatic insect could be used for classification of water bodies.
